# Supplementary material for: Hypomethylation of ATP1A1 Is Associated with Poor Prognosis and Cancer Progression in Triple-Negative Breast Cancer
Source: Cancers (Basel). 2024 Apr 25;16(9):1666. doi: 10.3390/cancers16091666 (PMC11083557; doi:10.3390/cancers16091666)
Supplement: Supplementary file 1 [file cancers-16-01666-s001.zip › cancers-2952688-supplementary.pdf]

Supplementary Figures

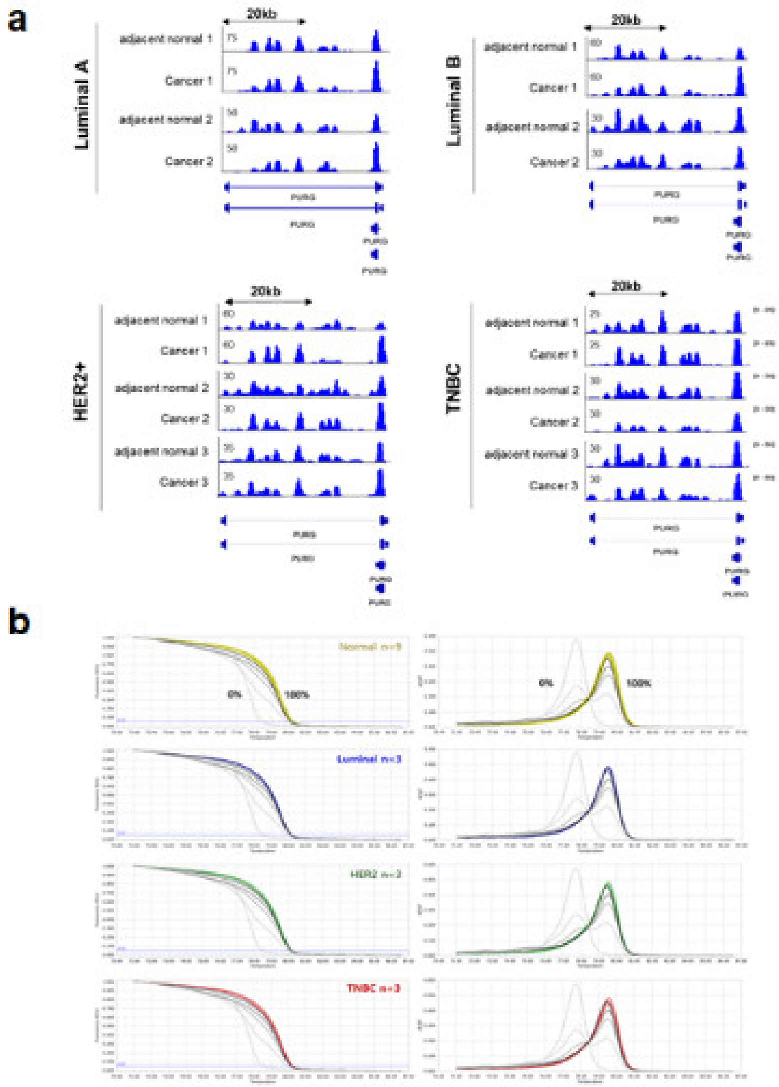

Supplementary Figure S1. Validation of the imprinting control region (ICR) of the PURG gene using methylation-sensitive high resolution melt (MS-HRM) analysis in different breast cancer subtypes and adjacent non tumor lesions. (A) Raw sequencing tag profiles of the imprinting control region (ICR) of the PURG locus on chromosome 8. Blue bars indicate that the gene body of the PURG gene had high peak calling in all breast samples. (B) Standard melting curves shown with a grey color gradient: 0%, light grey; 100%, dark grey. PURG melting curves overlapping the 100% control indicated full methylation in breast tumor and NTL samples.

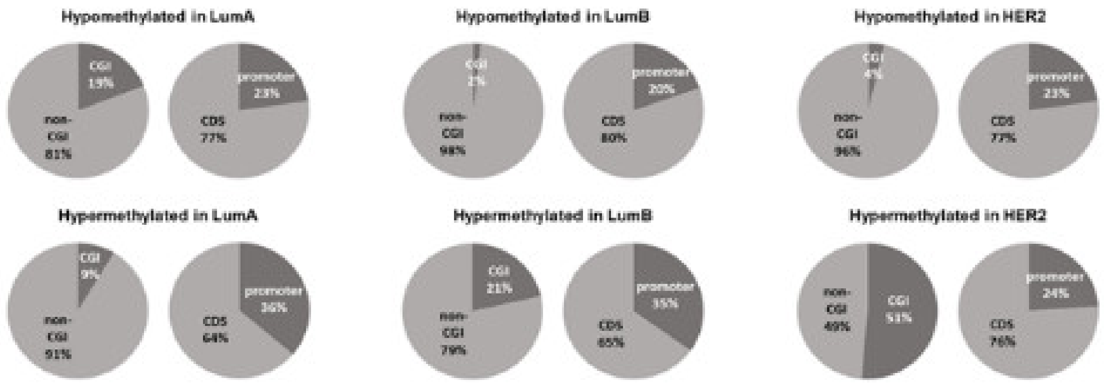

Supplementary Figure S2. Identification of DMR between breast tumor and non-tumor lesion in luminal A, luminal B, and HER2 subtypes. Pie charts representing the percentage of hyper- and hypomethylated DMRs that overlapped with the promoter and CDS or CGI and non-CGI.

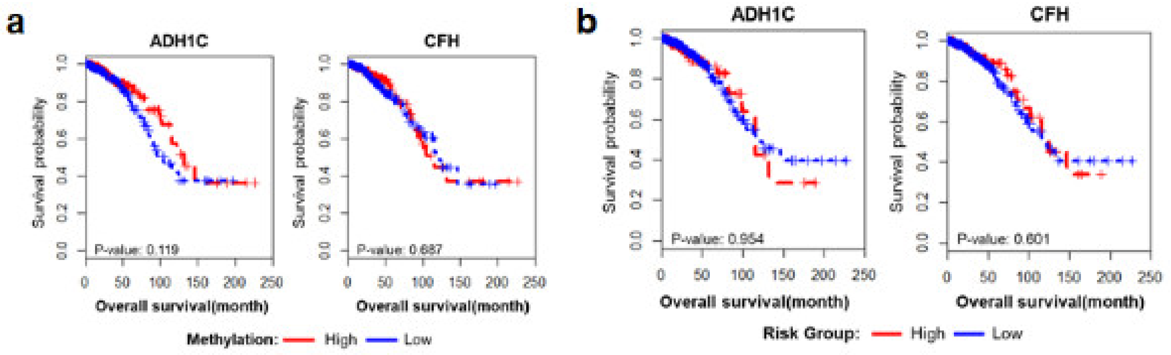

Supplementary Figure S3. Analysis of overall survival depending on ADH1C and CFH in basal-like breast cancer. (A) Overall survival analysis following methylation of ADH1C and CFH in basal-like breast cancer. (B) Classified samples to define high risk group of two genes were difference (ADH1C gene was defined by hypermethylated and low expressed samples, and CFH gene was defined by hypomethylated and low expressed samples). The others were classified into low risk group. We then performed a log-rank test to compare the Kaplan-Meier survival curves of these two groups.

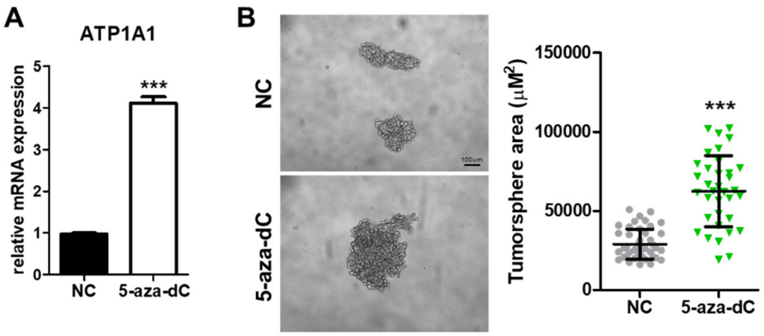

Supplementary Figure S4. Effect of demethylating agent on tumor sphere formation of MDA-MB-231. (A) Validation of ATP1A1 mRNA expression under treatment of 5-aza-dC (5uM). (B) Representative images of NC (negative control) or 5-aza-dC treated groups. Graph showing the sphere area of each group. ***P < 0.001.

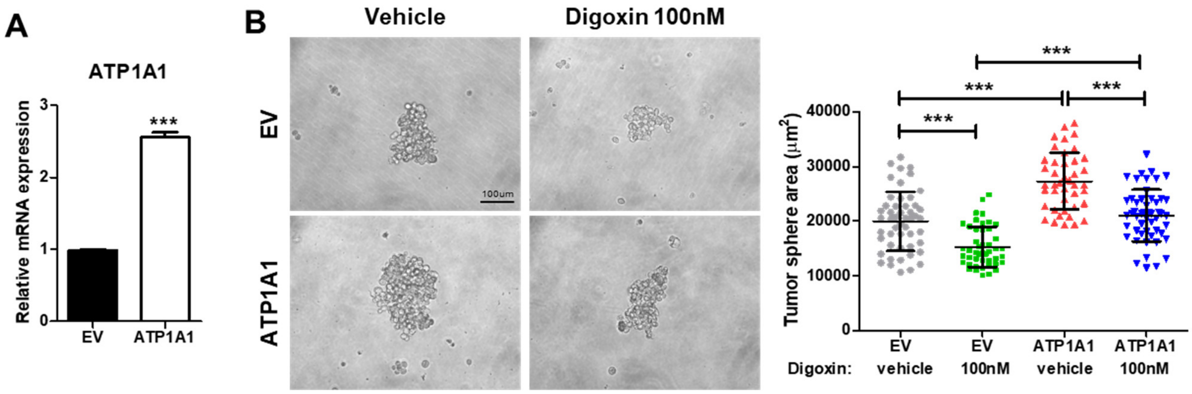

Supplementary Figure S5. Effect of digoxin treatment on control and ATP1A1 transfected MDA-MB-231 in tumorsphere condition. (A) Relative ATP1A1 mRNA expression in EV (empty vector) transfected and ATP1A1 transfected MDA-MB-231 cells. (B) Representative images of EV transfected MDA-MB-231, EV transfected MDA-MB-231 with digoxin (100nM), ATP1A1 transfected MDA-MB-231, and ATP1A1 transfected MDA-MB-231 with digoxin (100nM). Graph showing the sphere area of each group. ***P < 0.001.

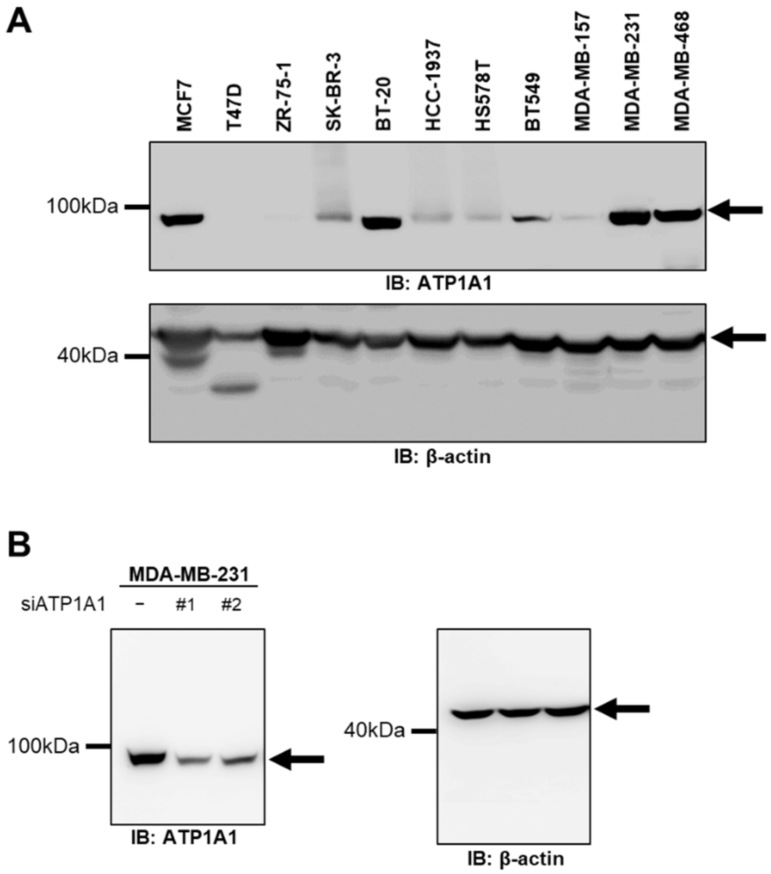

Supplementary Figure S6. Uncropped blots of western blot. (A) Uncropped blot of Fig 4D. (B) Uncropped blot of Fig 5A.

Supplementary Tables

Supplementary Table 1. Illumina sequencing read profiles

Sample	Total Reads sequenced	Number mapped to reference	Fraction mapped	Number unmapped to reference	Fraction unmapped	Suppressed multiple mapped reads	Fraction suppressed
LA N1	24,992,664	17,451,644	0.70	7,422,825	0.30	118,195	0.0047
LA C1	26,933,967	20,031,803	0.74	6,788,650	0.25	113,514	0.0042
LA N2	22,241,489	16,575,069	0.75	5,566,360	0.25	100,060	0.0045
LA C2	24,313,692	17,595,734	0.72	6,617,243	0.27	100,715	0.0041
LB N1	23,558,274	16,840,589	0.71	6,604,323	0.28	113,362	0.0048
LB C1	27,785,194	20,571,821	0.74	7,092,067	0.26	121,306	0.0044
LB N2	22,758,527	16,354,071	0.72	6,297,230	0.28	107,226	0.0047
LB C2	26,768,727	20,375,463	0.76	6,275,854	0.23	117,410	0.0044
HER2 N1	23,320,197	17,910,666	0.77	5,292,131	0.23	117,400	0.0050
HER2 C1	27,008,427	20,678,354	0.77	6,214,077	0.23	115,996	0.0043
HER2 N2	23,705,925	17,495,182	0.74	6,106,396	0.26	104,347	0.0044
HER2 C2	25,122,381	18,468,415	0.73	6,529,342	0.26	124,624	0.0050
HER2 N3	25,373,228	19,077,588	0.75	6,169,484	0.24	126,156	0.0050
HER2 C3	28,530,260	20,942,751	0.73	7,434,615	0.26	152,894	0.0054
TNBC N1	22,662,185	16,056,015	0.71	6,500,202	0.29	105,968	0.0047
TNBC C1	20,850,030	15,646,398	0.75	5,107,992	0.25	95,640	0.0046
TNBC N2	25,374,368	18,542,243	0.73	6,703,264	0.26	128,861	0.0051
TNBC C2	25,810,759	19,898,938	0.77	5,803,224	0.22	108,597	0.0042
TNBC N3	22,986,322	17,206,623	0.75	5,670,840	0.25	108,859	0.0047
TNBC C3	23,093,237	17,459,322	0.76	5,535,582	0.24	98,333	0.0043

Supplementary Table 2. Specific primers for PCR amplification of MS-HRM

Primer	Sense	Antisense
PURG MCA	TGTAGTTGAAGATGGAAAGAGTTAGG	ATAAAATCCCCTAAACAATCCTTCA
ATP1A1 MCA1	TGTGATGGGAAGAATTGTTATATTT	TTTCTCCACTACACTATTCAAAACC
